# The University of California-Los Angeles (UCLA) shoulder scale: translation, reliability and validation of a Thai version of UCLA shoulder scale in rotator cuff tear patients

**DOI:** 10.1186/s12891-022-05018-0

**Published:** 2022-01-18

**Authors:** Sorawut Thamyongkit, Thitiphol Wanitchanont, Kulapat Chulsomlee, Panithan Tuntiyatorn, Satetha Vasaruchapong, Chaiyanun Vijittrakarnrung, Nadhaporn Saengpetch

**Affiliations:** 1 Chakri Naruebodindra Medical Institute, Faculty of Medicine Ramathibodi Hospital, Mahidol University, Samut Prakan, Thailand; 2 Department of Orthopaedic surgery, Faculty of Medicine Ramathibodi Hospital, Mahidol University, Bangkok, Thailand

**Keywords:** UCLA, questionnaire, shoulder score, shoulder scale, rotator cuff

## Abstract

**Background:**

UCLA Shoulder Scale is a useful evaluation tool to assess the functional outcome of shoulder after treatments. It has been translated into several languages. The objectives of this study were to translate UCLA Shoulder Scale into Thai language and validate the translated version in patients with rotator cuff tear.

**Methods:**

This study consists of 2 phases: 1) Development of the Thai version of UCLA Shoulder Scale and 2) Validation of the translated version. The UCLA Shoulder Scale was translated into Thai according to the international guideline. Seventy-eight subjects with a mean age of 71 ± 11.5 took part in the study. All had shoulder pain and rotator cuff tear according to MRI from 2019 to 2020. Four patients were excluded due to incomplete questionnaires. The data from 21 patients whose symptoms in shoulder joint had not changed within 14 days were analyzed with the UCLA Shoulder Scale test-retest using intraclass correlation (ICC), Standard Error of Measurement (SEM) and Minimal Detectable Change (MDC). The Thai version of UCLA Shoulder Scale was compared to the validated Thai versions of American Shoulder and Elbow Surgeons (ASES), Western Ontario Rotator Cuff (WORC) and Shortened version of The Disability of the Arm, Shoulder and Hand (QuickDASH) shoulder scores.

**Results:**

Thai version of UCLA Shoulder Scale was developed following the guideline. Moderate to strong correlations were found using Spearman’s correlation coefficient between pain, function and total score of Thai version of UCLA Shoulder Scale. The reliability of total UCLA Shoulder Scale was excellent (ICC = 0.99, 95% CI 0.97–1.00), whereas agreement assessed with SEM and MDC (0.18 and 0.50 respectively) demonstrated a positive rating. The validity analysis of total UCLA Shoulder Scale (Thai version) showed moderate to strong correlations with total ASES, total WORC and QuickDASH (Thai versions). The Thai version of UCLA Shoulder Scale showed no floor and ceiling effects from the results.

**Conclusion:**

The Thai version of UCLA Shoulder Scale is a reliable and valid tool for assessing the function and disability of the shoulder in Thai patients who have rotator cuff tear.

## Background

Functional improvement is the most important goal after rotator cuff tear treatment. Pain and motion restriction range could lead to patients’ disability. Since reliability and accuracy are important for measuring tools, developing an appropriate one is needed to evaluate the patients undergoing/receiving shoulder treatment. During the past 20 years, various scoring systems have been used in clinical evaluation and research to represent treatment outcomes such as American Shoulder and Elbow Surgeons (ASES) shoulder score, Western Ontario Rotator Cuff (WORC), The Disability of the Arm, Shoulder and Hand questionnaire (DASH), shortened version of DASH (QuickDASH), and especially the University of California-Los Angeles (UCLA) Shoulder Scale [1–4]. A few Thai versions of shoulder scoring systems were used to evaluate shoulder function [5–7]. Reliability and accuracy are important for measuring tools.

UCLA Shoulder Score, originally published in 1981 in Clinical Orthopaedics and Related Research, was initially intended to assess clinical outcomes after total shoulder arthroplasty [8]. This assessment tool has later been thoroughly studied and widely used in the research area. Now that UCLA Shoulder Scale has been mainly used to evaluate outcomes in patients after surgery, it has been translated into many different languages such as Portuguese, Italian, Turkish and Polish [9–12]. However, to our best knowledge, it has not been translated into Thai language following the international guidelines, Linguistic Validation Manual for Patient-Reported Outcomes Instruments [13].

The objectives of this study were to develop the Thai version of UCLA Shoulder Scale from the English version and to evaluate its psychometric properties seeing that the Thai version of UCLA Shoulder Scale could be useful for clinical and research purposes concerning Thai population.

## Methods

**Fig. 1:**
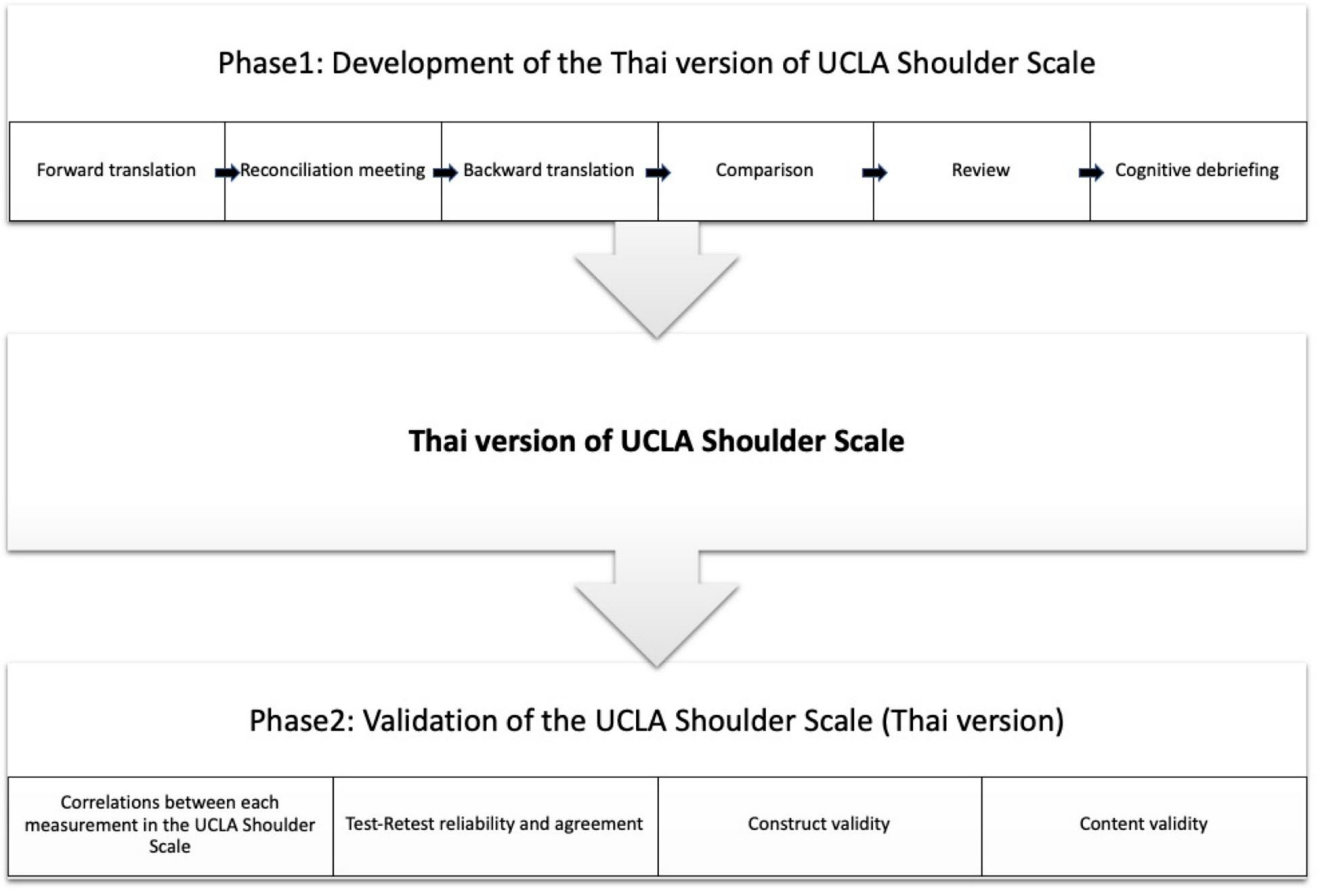
Flow chart of procedure of the study

Our study was divided into two phases (Fig. 1). The first step was to develop the Thai version of UCLA Shoulder Scale from the standard English version [14]. The permission for translation was granted from the publisher. The second was to validate the Thai version of UCLA Shoulder Scale and compare psychometric properties with common shoulder scoring systems including the Thai versions of ASES shoulder scale, WORC index and QuickDASH. The study was approved by the Institutional Review Board (IRB) committee of Mahidol University (IRB number MURA2020/323). Written informed consent was obtained from all individual participants included in the study.

UCLA Shoulder Scale contained two parts of questions: physician and patient sections. The physician section was based on physical examination. It consisted of two single-item sub-scales, which included “active forward flexion” (maximum of five points and completed by physicians), and “strength of forward flexion” (maximum of five points and completed by physicians). In contrast, the patient self-completed section of UCLA Shoulder Scale consisted of three single-item sub-scales, “pain” (maximum of ten points and completed by patients), “satisfaction” (maximum of five points and completed by patients), and “function” (maximum of ten points and completed by patients). Scores ranged from 0 to 35 with a score of 0 indicating worst shoulder function and 35 indicating best shoulder function.

### Phase1: Development of the Thai version of UCLA Shoulder Scale

The patient self-completed section of UCLA Shoulder Scale was translated and adapted in accordance with the guidelines [13]. The process comprised six stages:

1. Forward translation – Two independent English-Thai translations were prepared by two independent translators (ST and TW).
2. Reconciliation meeting – We arranged a meeting between 2 translators (ST and TW) and the senior author (NS) to analyzed both versions. Discrepancies between the two translations were addressed. At this stage, the team took an appropriate action and agreed on a joint version of the translation. This process is repeated until the meaning of the translated document is mutually agreed to be clear, concise and equivalent to the original.
3. Backward translation – The agreed version was translated backward into English. This stage was done by an independent professional English translator who was fluent in English and Thai.
4. Comparison -The original English version and the backward English version were compared. Following detailed analysis, the corrections were made by the team including backward translator and authors (ST, TW and NS). A further Thai version of UCLA Shoulder Scale was created.
5. Review - The latest version was reviewed by a team of expert orthopaedic surgeons (KC, PT, and SV). The team assessed the consistency of the original English version and the Thai version. Consistency was assessed via a 6-level scale from 0 to 5 (0 = inadequate, 5 = fully adequate). When questions were marked level 3 or lower, they were discussed by the team to make appropriate changes for the translation.
6. Cognitive debriefing - The clarity, understandability and acceptability of the Thai version of UCLA Shoulder Scale were tested on five Thai patients who had rotator cuff tear for a minimum period of 3 months. This group of patients filled out comprehensive assessment score, which was used to assess whether the given questions were fully comprehensible or not. Comprehension was assessed via a four-level scale (0 = totally incomprehensible, 3 = fully comprehensible). In cases where questions were considered incomprehensible, the assessing patients were asked to give the reasons for the lack of understanding. The group of authors (ST, TW and NS) analyzed and revised the questions to create the final Thai version of UCLA Shoulder Scale.

### Phase 2: Validation of the UCLA Shoulder Scale (Thai version): Tests for psychometric properties

Study participants were enrolled from the department of orthopaedics in a single university-based hospital. Seventy-eight subjects who were diagnosed with rotator cuff tear according to MRI and had failed from conservative treatment between 2019 and 2020 were eligible for the study. All patients had at least 6 months of consistent shoulder symptoms. They were above 18 years old, native Thai speakers who signed the informed consent to participate in the study. Exclusion criteria were applied with patients who had previous shoulder fractures or surgeries, osteoarthritis, shoulder dislocations, scapular fractures, clavicle or upper limb fractures; patients who had rheumatoid arthritis or neurological conditions; and patients who cannot fully understand nor signed the informed consent. Four patients were excluded due to incomplete questionnaires. Each patient was evaluated by both Thai versions of UCLA Shoulder Scale and WORC score pre-operatively. The study was evaluated only in patients who had rotator cuff tear because they had both pain and disability which need accurate evaluation tools. Test-retest reliability was also assessed in these 21 patients who completed the UCLA Shoulder Scale for two times. The interval between test and retest was 2 weeks.

### Internal consistency

The internal consistency of multi-item sub-scales was not assessed in UCLA pain and function sub-scales because these two sub-scales consisted of a single item. However, correlations between each measurement in the UCLA Shoulder Scale were evaluated using Spearman’s rank correlation coefficient (SCC).

### Test-Retest reliability and agreement

The intraclass correlation (ICC) was used to assess the reliability of UCLA Shoulder Scale. This was calculated from the group of 21 patients who had completed the UCLA Shoulder Scale for two times. According to the guidelines from the literature, we assumed a positive rating for reliability when the ICC is ≥0.70 [13].

Agreement is the property related to the absolute measurement error by the instrument when two or more measurements repeated in the same condition. Standard Error of Measurement (SEM) and the Minimal Detectable Change (MDC) were calculated to assess the agreement [15]. SEM was calculated using the formula: SEM = SD √(1-R), where SD represents Standard Deviation of the sample and R represents the reliability parameter (ICC). MDC was calculated using the formula: MDC=SEMx1.96× √2, where 1.96 derives from the 0.95% CI of no change, and √2 shows two measurements assessing the change. We gave a positive rating for agreement if the MDC was smaller than Minimal Important Change (MIC). Additionally, we defined MIC = 1, which was the smallest number scale difference in this scoring system [16]. The data calculation was done in the group of 21 people who had completed the UCLA Shoulder Scale for two times.

### Content validity

Content validity refers to the degree that the instrument covers the content that it is supposed to measure. Indexes of Item-Objective Congruence (IOC) was used to evaluate content validity of Thai version of UCLA Shoulder Scale. The IOC of each item was calculated using summation of score from each orthopaedic surgeons (ST, TW, KC, PT and SV) divided by the number of surgeons.

Floor or ceiling effect was considered to be present if more than 15% of the respondents achieved the lowest or highest possible score, respectively [15]. Floor and ceiling effects were calculated from the group of 74 patients for UCLA Shoulder Scale, ASES shoulder score, WORC and QuickDASH.

### Construct validity

Construct validity was evaluated to ensure that scores in Thai version of UCLA Shoulder Scale is consistent with the concepts that are being measured [15]. To evaluate the construct validity, we analyzed the correlation between the Thai version of UCLA Shoulder Scale and Thai version of ASES Shoulder Scale, WORC and QuickDASH [7, 17, 18]. Construct validity of the Thai version of UCLA Shoulder Scale was evaluated by the Spearman’s correlation coefficient (SCC). Correlation coefficients: r < 0.30 = low, 0.30 < r < 0.70 = moderate, and r > 0.70 = high, were used to assess the validity [19].

### Statistical analysis

The level of statistical significance was assumed a priori at α < 0.05. Shapiro-Wilk test show that the results had a non-normal distribution. Spearman’s correlation was used to evaluate the correlation between each measurement in UCLA Shoulder Scale. The test-retest reliability was analyzed using intraclass correlation (ICC), two-way random-effects model [20]. Based on a systematic review study [21], appropriate sample size is at least 15 subjects for our test-retest reliability (the sample size of about 5 times the number of items). The internal consistency was measured using Cronbach’s α. The sample size was based on the general recommendations of Altman of at least 50 subjects in a method comparison study [22]. Statistical analyses were performed using SPSS 11.0 for Windows (SPSS, Chicago, IL, USA). A *p*-value < 0.05 was considered statistically significant.

## Results

### Phase 1: Development of the Thai version of UCLA Shoulder Scale

According to the guidelines [13], the translation and adaptation to develop the Thai version of UCLA Shoulder Scale was carried out in six stages.

- Stage I: In this stage, two orthopaedic surgeons (ST and TW) independently translated the UCLA Shoulder Scale [14] from English into two Thai versions. The translators used Thai words and sentences that had the most equivalent or the closest meaning to the original English version.
- Stage II: A team of two translators and co-author (NS) analyzed and adapted the Thai version for better consistency and comprehension of the questionnaire. In this stage, ten words/sentences in both physician and patient sections were adapted.
- Stage III: A professional English translator backward translated the agreed version from stage II into English.
- Stage IV: The backward translation version was compared with the original English version by the team of authors and a professional translator. The discrepancies were addressed and then corrected according to the team’s agreement. For example, we used the term “anti-inflammatory drug or Tylenol” instead of “Salicylate” because this term was commonly used for medication for mild to moderate pain in Thailand. (Table 1)
- Stage V: The consistency of Thai version was assessed by the team of five orthopaedic surgeons, who currently practice in patients with shoulder pain. The assessment was made via a 6-level scale: when the consistency level was marked 3 or lower, the words used in the questionnaire were corrected according to the agreement of authors and orthopaedic surgeons.
- Stage VI: The Thai version of UCLA Shoulder Scale was tested with a group of five Thai patients who were diagnosed with rotator cuff tear and had been suffering from it for at least 6 months. The group was composed of three women and two men aged from 62 to 75 years old. After the analysis of the answers was received from the group of five patients, an average comprehensive assessment score of 2.41 was obtained.

**Table 1:** The changes made to the UCLA Shoulder Score (Thai version) during Stage IV

No.	Original English version (OV)	Thai version after joint translation (TV)	English version after backward translation (BT)	Thai version corrected after backward translation (CV)
3	Non or little at rest, present during light activities; salicylates frequently	ไม่เจ็บปวดหรือปวดเล็กน้อยเมื่อพัก ปวดเมื่อทำกิจกรรมเบาๆ	Don’t feel pain or feel only a little at rest, feel pain at light activities; use salicylates frequently	ไม่เจ็บปวดหรือเจ็บปวดเล็กน้อยเมื่อพัก เมื่อทำกิจกรรมเบาๆ ต้องใช้ยาลดการอักเสบหรือไทลินอลบ่อยๆ
4	present during heavy or particular activities only; salicylates frequently		Feel pain during heavy or particular activities only; use salicylates frequently	เจ็บปวดเมื่อทำกิจกรรมหนักๆหรือกิจกรรมบางประเภทเท่านั้น ต้องใช้ยาลดการอักเสบหรือไทลินอลบ่อยๆ
11	slight restriction only; able to work above shoulder level	ใช้งานได้เกือบปรกติ / ยกแขนเหนือศีรษะได้	Have few limitations-able to work above shoulder level	มีข้อจำกัดเพียงเล็กน้อยเท่านั้น สามารถยกแขนทำกิจกรรมเหนือระดับไหล่ได้

### Phase 2: Validation of the UCLA Shoulder Scale (Thai version)

Seventy-four participants completed the Thai version of UCLA Shoulder Scale as well as the Thai versions of ASES and QuickDASH questionnaires. Their demographic data were shown in Table 2. The correlation between each measurement in UCLA Shoulder Scale had moderate to strong correlations (0.43–0.76) (Table 3).

**Table 2:** Demographic characteristics and baseline UCLA Shoulder Scale of patients

Characteristics	n (%)	mean (SD)
Gender		
Male	40 (54.1%)	
Female	34 (45.9%)	
Age (years)		71.0 (11.5)
Duration of shoulder pain		10.8 (5.1)
UCLA		
Pain dimension		5.1 (2.9)
Function dimension		4.3 (2.3)
Satisfaction dimension		3.0 (2.4)
Forward flexion (degrees)		115.5 (39.6)
Forward flexion score		3.5 (1.3)
Strength of forward flexion		4.2 (0.9)
Total UCLA		19.9 (6.8)

**Table 3:** Spearman’s correlation coefficient (SCC) between each measurement

UCLA score	Pain	Function	Forward flexion	Strength of forward flexion	Satisfaction	Total UCLA
Pain	1.00	0.50	0.03	0.27	0.43	0.76
Function	0.50	1.00	0.46	0.58	0.22	0.72
Forward flexion	0.03	0.46	1.00	0.84	0.09	0.49
Strength of forward flexion	0.27	0.58	0.84	1.00	0.25	0.68
Satisfaction	0.43	0.22	0.09	0.25	1.00	0.66
Total UCLA	0.76	0.72	0.49	0.68	0.66	1.00

The UCLA Shoulder Scale was compared between test 1 and test 2 (re-test) in a group of 21 patients. There was a significant difference of total UCLA Shoulder Scale (*p* < 0.05). However, the difference was small in relation to the initial result, which was − 0.62. The ICC for the total UCLA was 0.99 and the domains ranged between 0.93 and 0.99, suggesting a high consistency of the results of both tests for all UCLA domains.

The Standard Error of Measurement (SEM) and Minimal Detectable Change (MDC) for total UCLA were 0.18 and 0.50 respectively. SEM for each domain ranged from 0.07 to 0.33 and MDC ranged from 0 to 0.92, which were smaller than Minimal Important Change (MIC). Based on these results, there was no significant change of state occurred in the study group (Table 4).

**Table 4:** Assessment of reliability using ICC and assessment of agreement using SEM and MDC for UCLA Shoulder Scale

UCLA score	change test 2 vs. test 1				ICC (95% CI)	SEM	MDC
	average	median	SD	*p* value			
Pain	−0.2	0.0	0.9	0.329	.982 (.916–.985)	0.12	0.32
Function	0.0	0.0	1.3	0.867	.933 (.835–.973)	0.33	0.92
Forward flexion	0.2	0.0	1.1		1		0.00
Strength of forward flexion	0.0	0.0	0.0		1		0.00
Satisfaction	−0.2	0.0	1.1	0.329	.938 (.848–.974)	0.07	0.19
Total UCLA	−0.6	0.0	1.6	0.091	.987 (.967–.995)	0.18	0.50

Indexes of Item-Objective Congruence (IOC) was used to evaluate content validity of patient self-completed section in Thai version of UCLA Shoulder Scale (Table 5). Floor and ceiling effects were not presented (< 15%) in a group of 74 patients for UCLA, ASES, WORC and QuickDASH. (Table 6).

**Table 5:** Indexes of Item-Objective Congruence (IOC) of the Thai version of UCLA Shoulder Scale (Patient self-completed section)

Items		IOC
P1	Present always and unbearable; strong medication frequently	0.6
	ตลอดเวลาและทนไม่ได้ ต้องใช้ยาขนานแรงบ่อยๆ	
P2	present always but bearable; strong medication occasionally	0.6
	ตลอดเวลาแต่ทนได้ ใช้ยาขนานแรงเป็นครั้งคราว	
P3	Non or little at rest, present during light activities; salicylates frequently	0.6
	ไม่เจ็บปวดหรือเจ็บปวดเล็กน้อยเมื่อพัก เมื่อทำกิจกรรมเบาๆ ต้องใช้ยาลดการอักเสบหรือไทลินอลบ่อยๆ	
P4	present during heavy or particular activities only; salicylates frequently	0.6
	เจ็บปวดเมื่อทำกิจกกรมหนักๆหรือกิจกรรมบางประเภทเท่านั้น ต้องใช้ยาลดการอักเสบหรือไทลินอลบ่อยๆ	
P5	Occasional and slight	1
	เจ็บปวดเพียงเล็กน้อยและเป็นครั้งคราว	
P6	None	1
	ไม่มีความเจ็บปวดเลย	
F1	Unable to use limb	0.8
	ไม่สามารถใช้งานแขนข้างที่เป็นได้เลย	
F2	Only light activities possible	0.8
	ใช้งานเบาๆได้เท่านั้น	
F3	Able to do house work or most activities of dialy living	1
	ทำกิจวัตรประจาวันส่วนใหญ่และงานบ้านได้	
F4	Most housework, shopping and driving possible; able to do hair and to dress and undress, including fastening brassiere	0.6
	ทำงานบ้านส่วนใหญ่ได้ รวมถึงสามารถไปจับจ่ายและขับรถได้ สามารถทำผมเอง แต่งตัวและถอดเสื้อผ้าเอง รวมถึงติดตะขอยกทรงเองได้	
F5	slight restriction only; able to work above shoulder level	0.8
	มีข้อจำกัดเพียงเล็กน้อยเท่านั้น สามารถยกแขนทำกิจกรรมเหนือระดับไหล่ได้	
F6	Normal activities	1
	ใช้งานได้ปรกติ	
S1	Satisfied and better	0.8
	พึงพอใจและรู้สึกดีขึ้น	
S2	Not satisfied	0.8
	ไม่พึงพอใจอาการ

**Table 6:** Floor, ceiling and skewness of Thai version of UCLA Shoulder Scale

Shoulder Scores	N	Mean (SD)	SD	Floor, n (%)	Ceiling, n (%)	Skewness (SE)
Total UCLA	74	19.9189	6.86615	0 (0%)	1 (1.4%)	0.167 (0.279)
Total ASES	74	55.991	21.39702	0 (0%)	1 (1.4%)	−0.184 (0.279)
WORC	74	58.8467	24.16217	0 (0%)	0 (0%)	−0.295 (0.281)
QuickDASH	74	39.0387	23.0239	1 (1.4%)	0 (0%)	0.309 (0.279)

**Fig. 2:**
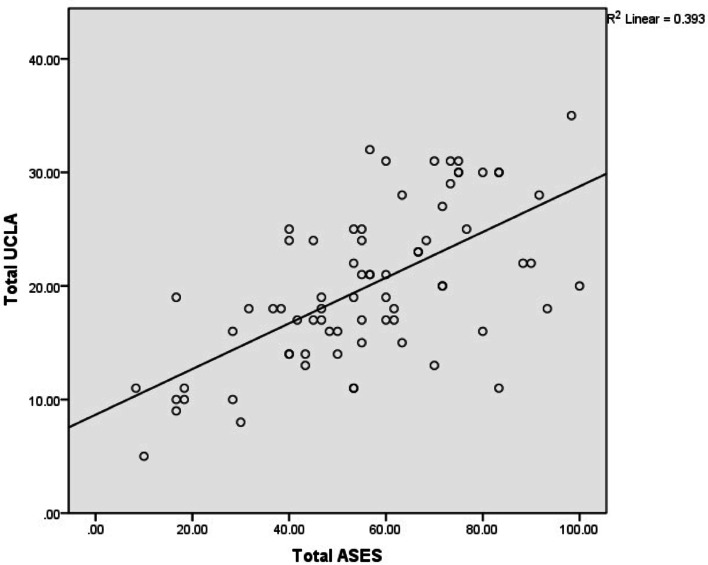
A scatter plot of total UCLA vs. total ASES scores. Spearman’s correlation Coefficient = 0.610, *p* < 0.01

**Fig. 3:**
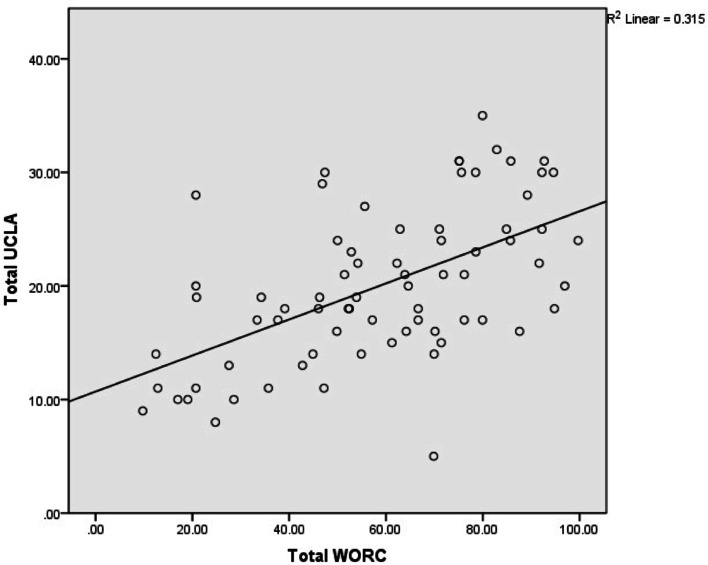
A scatter plot of total UCLA vs. total WORC scores. Spearman’s correlation Coefficient = 0.572, *p* < 0.01

**Fig. 4:**
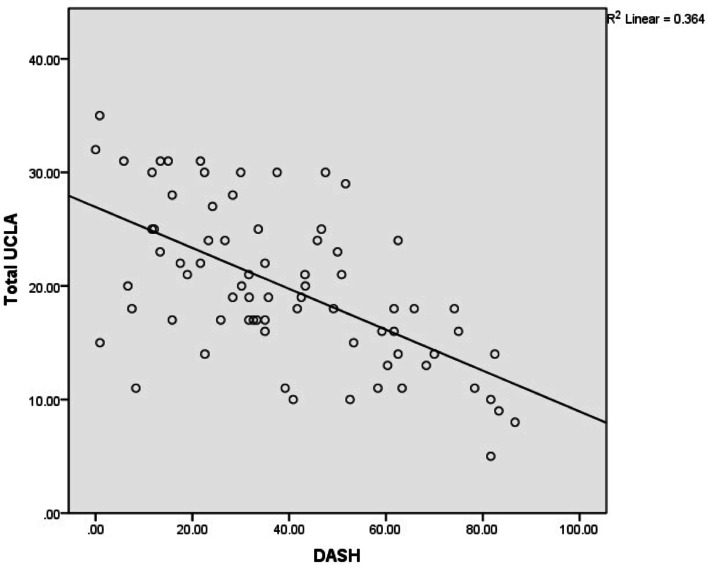
A scatter plot of total UCLA vs. QuickDASH scores. Spearman’s correlation Coefficient = − 0.585, *p* < 0.01

The construct validity of the Thai version of UCLA Shoulder Scale was assessed using Spearman’s correlation coefficient. The total UCLA Shoulder Scale moderately correlated with total ASES, WORC and QuickDASH scores (*p* < 0.01). There are moderate correlations between UCLA domain of pain, ASES domain of pain, WORC domain of symptoms and QuickDASH. Also, there are moderate correlations between UCLA domain of function, ASES domain of function, WORC domain of work and QuickDASH (Table 7). Figures 2, 3 and 4 showed a scatter plot of total UCLA vs total ASES, total WORC and QuickDASH, respectively.

**Table 7:** Spearman’s correlation coefficient between UCLA Shoulder Scale and reference questionnaires (ASES, WORC and QuickDASH)

Indices	UCLA score											
	Pain	***p***-value	Function	***p***-value	Satisfaction	***p***-value	Forward flexion	***p***-value	Strength of forword flexion	***p***-value	Total UCLA	***p***-value
**ASES score**												
Pain	−0.536	< 0.01	− 0.39	< 0.01	− 0.365	< 0.01	0.03	0.8	− 0.151	0.201	− 0.435	< 0.01
Function (total ADL)	0.452	< 0.01	0.672	< 0.01	0.276	<.005	0.327	< 0.01	0.403	< 0.01	0.587	< 0.01
Total ASES	0.599	< 0.01	0.626	< 0.01	0.384	< 0.01	0.156	0.187	0.313	< 0.01	0.610	< 0.01
**WORC**												
Physical symptoms	−0.647	< 0.01	−0.39	< 0.01	− 0.331	< 0.01	− 0.78	0.515	− 0.16	0.18	− 0.536	< 0.01
Sports/ recreation	−0.213	< 0.01	− 0.327	< 0.01	−0.239	<.005	−191	1.09	−0.185	0.12	−0.313	< 0.01
Work	−0.328	< 0.01	−0.559	< 0.01	− 0.341	< 0.01	−0.188	0.114	−0.266	<.005	−0.495	< 0.01
Lifestyle	−0.571	< 0.01	−0.566	< 0.01	− 0.354	< 0.01	−0.134	0.263	−0.265	<.005	−0.573	< 0.01
Emotions	−0.521	< 0.01	−0.374	< 0.01	− 0.343	< 0.01	0.26	0.826	−0.111	0.353	−0.432	< 0.01
Total WORC	0.542	< 0.01	0.542	< 0.01	0.398	< 0.01	0.157	0.187	0.242	<.005	0.572	< 0.01
**QuickDASH**	−0.529	< 0.01	− 0.565	< 0.01	− 0.263	<.005	− 0.319	< 0.01	− 0.388	< 0.01	−0.585	< 0.01

## Discussion

The study demonstrated the process of translation and validation of Thai language version of UCLA shoulder score. The results show good validity and reliability of the translated version. Furthermore, in terms of construct and convergent validity, there were moderate to strong correlations between each item in UCLA Shoulder Scale. The scale also had moderate correlation with ASES regarding pain vs. pain dimension (SCC = − 0.536, *p* < 0.01). These results are comparable to the previous studies [23, 24], which showed significant correlation of the original UCLA Shoulder Scale with other shoulder scoring systems including ASES, DASH, Oxford Shoulder Score, and Constant Shoulder Score.

The reliable and comprehensible questionnaire is needed for effective evaluation of patients’ functional status and treatment outcomes. UCLA Shoulder Scale itself has some concerns regarding double-barreled items and allocated points of each item, which might cause some difficulties for respondents to pick an appropriate answer for different items. Despite the fact that UCLA Shoulder Scale was developed at the time when the modern psychometric test had not yet been established, it has still been widely used for functional evaluation of the shoulder in clinical practices and research. There are studies on psychometric properties of other language versions of UCLA Shoulder Scale. The results show comparable reliability and validity to the original English version [9, 10, 12]. UCLA Shoulder Scale is accepted to be a useful tool because it is relatively quick and easy for respondents to complete the questionnaire compared to other tools.

UCLA Shoulder Scale has a patient self-completed section which needs the patients to understand each question comprehensively. For this reason, the questionnaire for patients is usually translated into local language. However, to standardize the questionnaire, the translation process comprised multiple steps. This study focused on the process of literal translation of UCLA Shoulder Scale into Thai language. Both translation of questionnaire into Thai and backward translation were performed complying with international guidelines [13]. We assigned orthopaedic surgeons to do the translation in stage I of development because they understand and are familiar with the language used in physician-patient conversation. To minimize the translation error, a professional English language translator, orthopaedic surgeons and patients suffering from rotator cuff tear were involved in the translation process. In the process of questionnaire development, three items were changed during phase IV. These issues were identified and resolved in the course of the team’s discussions. In the process of content validation, we assessed by examining floor and ceiling effects and skewness of distribution. In the previous studies on translating other shoulder scoring systems into Thai language, there was a good content validity with negligible floor and ceiling effects [5, 6, 25]. However, the Thai version of UCLA Shoulder Scale in our study had a good content validity with no floor and ceiling effects as demonstrated in Table 4. In this study, the floor and ceiling effects ranged between 0 to 1.4% and skewness ranged between − 0.295 and 0.309.

The strength of this study is that it is the first Thai language version of the UCLA Shoulder Scale which was translated in compliance with the international guidelines [13]. The study also provided the evidence of accuracy and psychometric properties of the Thai version of UCLA Shoulder Scale. Nevertheless, our study still had some limitations. First, the study had a relatively small number of participants completing the test-retest reliability. The larger sample size could increase the reliability and accuracy of the study result. Second, the participants were enrolled in a single university-based institute which might not represent the entire Thai population. Despite the mentioned limitations, the study showed that the Thai version of UCLA Shoulder Scale had fair to good correlation with other scoring systems. This correlation level is similar to the English version of UCLA Shoulder Scale [16, 23, 26].

## Conclusion

The Thai language version of UCLA Shoulder Score constitutes a valuable tool to evaluate shoulder function in patients who have rotator cuff tear. The study demonstrated good validity and reliability of the Thai version of UCLA Should Scale. This shoulder functional scoring system could be the useful evaluating tool in the aspects of further clinical and research use because of its clarity and comprehensibility for Thai patients.

## Abbreviations

UCLA: The University of California-Los Angeles
MRI: Magnetic Resonance Imaging
ICC: Intraclass correlation coefficient
SEM: Standard Error of Measurement
MDC: Minimal Detectable Change
MIC: Minimal Important Change
ASES: The American Shoulder and Elbow Surgeons
WORC: The Western Ontario Rotator Cuff Index
DASH: The Disabilities of the Arm, Shoulder and Hand
